# Random Forest-Assisted Widely Targeted Lipidomic Reveals Differences in Tan Lamb Meat Quality in Different Regions

**DOI:** 10.3390/foods14234046

**Published:** 2025-11-26

**Authors:** Qi Yang, Chongxin Liu, Muxuan Xu, Minghui Gu, Le Xu, Shaobo Li, Xiaochun Zheng, Dequan Zhang, Li Chen

**Affiliations:** Key Laboratory of Agro-Products Quality and Safety Control in Storage and Transport Process, Institute of Food Science and Technology, Chinese Academy of Agriculture Sciences, Ministry of Agriculture and Rural Affairs, Beijing 100193, China

**Keywords:** Tan lamb, meat quality, random forest, lipidomic, different regions

## Abstract

This study aims to elucidate the quality differences among Tan lamb from three major production regions and investigate the influence and regulation of lipids on regional sheep meat quality. The Ningxia Tan lamb exhibited higher pH values, lower lightness and yellowness, better water-holding capacity and more polyunsaturated fatty acids compared to the other regions. GC-IMS revealed different flavor profiles of Tan lamb from different regions. A total of 1080 lipids across 41 lipid subclasses were identified, with 10 lipid molecules, including PC (16:0_16:1), Carnitine C3:0 and Carnitine C5:1, serving as key differentiators among the regions, as determined by the Random Forest method. Correlation analysis revealed strong associations between acyl lipid characteristics and pH, lightness and centrifugal loss, while glycerophospholipid characteristics were significantly correlated with basic nutritional indices. Lipid metabolic pathway analysis indicated that thermogenesis, glycerophospholipid metabolism and metabolic pathways as crucial for Tan lamb growth, serving as major pathways for distinguishing different regional Tan lamb. These findings indicate that origin influences lamb quality and lipid composition and that a relationship may exist between lamb quality and lipid variations. This provides a comprehensive understanding of how lamb quality is formed and contributes to future identification of lamb origins, as well as control and enhancement of meat quality.

## 1. Introduction

Lamb occupies an important position in the global food market. Among them, Tan lamb is especially favoured for its tender texture and rich nutritional content [1]. As a distinctive and superior livestock breed indigenous to China, Tan sheep are predominantly bred in Yanchi County of Ningxia, with their distribution also extending to neighboring regions such as parts of Gansu, Shaanxi, and Inner Mongolia [2]. Due to variations in rearing practices, geographical environments, and climatic conditions, the quality of Tan lamb differs across these regions. Understanding these regional quality differences is crucial for enhancing the market competitiveness of Tan lamb and informing consumer choices.

Meat quality is a key indicator of the value of meat and includes aspects such as pH, water retention, meat colour, flavor, tenderness, and fat content [3]. These indicators significantly influence consumers preferences. Lipids, as one of the main components of meat, play a critical role in determining meat quality. Lipid molecules serve as important flavour precursors, generating volatile compounds through lipid oxidation and the Maillard reaction, which determine the final flavour of the meat [4]. Different levels and ratios of lipids can impart various flavours and textures to meat. For instance, 4-alkyl-branched fatty acids are considered the primary source of lamb’s off-odor [5], differences in lipids produced by feed may result in unique lamb flavors [6]. Moreover, there are potential correlations between lipid content and meat colour, tenderness, and chewiness. Therefore, significant differences in lipid content and composition among foods from different regions may be key factors affecting Tan lamb quality [7].

Lipidomic, an emerging technology, provides a comprehensive understanding of lipid profiles and has been widely applied in meat science fields such as flavour development [8], breed identification [9], and quality comparison [10]. Applying lipidomic to study Tan lamb from different regions can offer deep insights into their lipid differences and metabolic characteristics. Furthermore, the random forest machine learning algorithm has demonstrated excellent performance in screening for potential biomarkers [11], offering a new method for identifying key markers in Tan lamb. Through the random forest algorithm, crucial lipid molecules affecting the quality of Tan lamb from various regions can be identified from extensive lipid data. This provides a scientific basis for understanding lipid differences and quality control in Tan lamb.

Preliminary research combining machine learning and lipidomics has enabled the origin identification of Yanchi Tan lamb [12]. Therefore, this study aims to elucidate the potential impact of regional variations in meat quality characteristics and lipid profiles on the quality and metabolism of Tan lamb, providing valuable reference for origin identification, meat evaluation, and quality control. We investigated the differences in meat texture, fatty acid composition, meat quality, and flavor among Tan lamb from three distinct regions. By combining widely targeted lipidomic with the random forest machine learning algorithm, the study revealed the lipids responsible for the quality differences in Tan lamb from different regions. Additionally, the research explored the relationship between meat quality differences and characteristic lipids in Tan lamb from different regions, as well as the key metabolic pathways involved in the formation of characteristic lipids.

## 2. Materials and Methods

### 2.1. Chemicals and Reagents

Lipid standards were purchased from Sigma-Aldrich or Avanti Polar Lipids (Alabaster, AL, USA). Methanol, acetonitrile Methyl-tert-butyl ether (MTBE) and isopropanol were procured from Merck (Darmstadt, Germany). Ammonium formate and dichloromethane were purchased from Thermo Fisher Scientific Inc. (Waltham, MA, USA). Formic acid was obtained from Sigma-Aldrich. All other chemical reagents used were of analytical grade.

### 2.2. Sample Collection

A total of 18 samples of Tan lamb knuckle meat were collected from Yanchi, Ningxia (NX); Jingyuan, Gansu (GS); and Ertokqianqi, Inner Mongolia (IM) (*n* = 6 for each). All samples were obtained from the carcasses of the same batch of slaughtered lambs with an average weight of 18.12 ± 1.29 kg (6 months old, male). After the company’s employees finish slaughtering, the Tan lamb carcasses are stored in a 4 °C de-acidification bank for 24 h post-mortem aging. The knuckle meat was collected, with visible fat and connective tissue removed, and approximately 1 g of muscle sample was weighed and placed into a cryotube for lipidomics assay. The cryotube containing the sample was immersed in liquid nitrogen for 30 min, shipped back to the laboratory on dry ice, and stored at −80 °C. The remaining meat samples were stored at −20 °C for other meat quality indicators.

### 2.3. Meat Quality Determination

The pH value of the lamb was measured using a calibrated testo 205 portable pH meter (Testo, Germany). The lightness (L*), redness (a*), and yellowness (b*) of the meat samples were determined using a calibrated CM-600d chromatic meter (Konica Minolta Investment Co., Tokyo, Japan). With a slight modification of the method of Xu et al. [13], the freshly cut side of the knuckle meat was placed facing upwards, and the lens of the colorimeter was placed vertically on the meat surface, avoiding the intramuscular fat and connective tissues, and each sample was measured three times, and the results were averaged. The determination of cooking loss was based on the method of Wang et al. [14] with slight modifications: approximately 55 g of meat was placed in a cooking bag, and a thermometer probe was inserted from top to bottom into the center of the meat. The packaged meat was then placed in a water bath at 72.0 °C. When the center temperature of the meat reached 70.0 °C (approximately 15 min), the meat sample was removed and cooled under running water for 30 min use absorbent paper to blot dry the surface juices of the meat piece before weighing. The percentage of weight loss before and after steaming relative to its original weight is the steaming loss.

Centrifugal loss was determined as follows: a 10.0 g meat sample was wrapped in qualitative filter paper and placed in a 50 mL centrifuge tube containing a 6 cm piece of absorbent cotton. The sample was centrifuged at 14,000× *g* for 10 min at 4 °C. After centrifugation, the meat sample was weighed without the filter paper and the percentage weight loss relative to the original weight represented the centrifugal loss.

The meat samples used for cooking loss measurement were stored overnight at 4 °C, equilibrated at room temperature for 0.5 h, and cut into meat columns of 2 cm × 1 cm × 1 cm along the muscle fibres. The shear force was measured using C-LM4 tenderness meter (Northeast Agricultural University, Harbin, Heilongjiang Province, China). Meat blocks after determination of cooking loss were equilibrated at room temperature for 0.5 h after overnight (about 12 h) in a refrigerator at about 4 °C, surface juices were drained with qualitative filter paper, and meat columns of 2 cm long, 1 cm wide and 1 cm high were divided along the direction of the muscle fibers, and meat columns were sheared with an instrument in the direction of the vertical muscle fibers, and the measurements were carried out in six copies and the results were averaged. The contents of crude fat, protein, and moisture in the meat samples were determined using the Soxhlet extraction method, the Kjeldahl method, and the direct drying method, respectively. All measurements were carried out in triplicates.

### 2.4. Fatty Acid Determination

The determination of fatty acids was slightly modified based on the method described by Xu et al. [13]. A 0.1 g dry sample powder was weighed and mixed with 3.8 mL of a CHCl_3_/CH_3_OH/H_2_O (1:2:0.8, *v*/*v*/*v*) solution. The mixture underwent ultrasonic extraction at 4 °C for 5 min, repeated three times. The resulting lipid sample was obtained via rotary evaporation. Next, 2 mL of NaOH/methanol/water (1:16:2, *w*/*v*/*v*) was added for saponification at 60 °C for 2 h. Then, 2 mL of CHCl_3_/n-hexane (1:4, *v*/*v*) was added and shaken for 2 min, repeated three times, to obtain saponified lipids. A 0.5 mL 10% boron trifluoride-methanol solution was added, and esterification was performed at 60 °C for 1 h. Fatty acid methyl esters (FAMEs) were extracted with 2 mL n-hexane and 3 mL CHCl_3_/n-hexane (1:4, *v*/*v*), and the supernatant was dried under nitrogen. The FAMEs were redissolved in 0.5 mL n-hexane and analyzed using an Agilent 7890 B-7000 C GC-MS system equipped with a CD-2560 capillary column (100 m × 250 μm × 0.2 μm, CNW, Frankfurt am Main, Germany). The temperature program and GC-MS parameters were set according to the method of Ran et al. [15].

Standard solutions at different concentrations of 0.01, 0.02, 0.05, 0.1, 0.2, 0.5, 1, 2, 5, 10, 20, and 50 μg/mL were prepared, and the chromatographic peak intensity data corresponding to each concentration were obtained. The external standard concentration was plotted on the *x*-axis and the external standard peak area on the *y*-axis to generate the standard curve (Table S2).

### 2.5. Volatile Compound Determination

A gas chromatography-ion mobility spectroscopy (GC-IMS) (FlavourSpec^®^, G.A.S., Dortmund, Germany) was used as described by Chen et al. [16] with slight modifications. A 5 g sample was placed in a headspace vial and incubated at 45 °C for 20 min, then 500 μL (85 °C) was extracted using an automated headspace sampling system. High-purity nitrogen was used as the carrier gas, and the separation was performed using MXT-5 (15 m × 0.53 mm × 1.0 μm, Restek, Bellefonte, PA, USA) at 60 °C. The separation was carried out using an automated headspace sampling system. The carrier gas flow rate was set as follows: 0–2 min: 2.0 mL/min, 2–10 min: linearly increasing to 10 mL/min, 10–20 min: linearly increasing to 100 mL/min, and 20–25 min: linearly increasing to 150 mL/min. Tritium source (^3^H) was used as the ionization source, and the detection was performed in the positive ion mode at 45 °C.

### 2.6. Lipid Extraction and Data Analysis

Lipids were extracted by MTBE method [17]. To 20 mg of sample, 1 mL of lipid extraction solution containing an internal standard (MTBE:methanol = 3:1, *v*/*v*) and 200 μL of water were added and homogenized. The mixture was centrifuged at 13,500× *g* for 10 min at 4 °C. Then, 200 μL of the supernatant was dried and reconstituted in 200 μL of reconstitution solution (acetonitrile:isopropanol = 1:1, *v*/*v*) and centrifuged again at 13,500× *g* for 3 min. Subsequently, 2 μL of the sample was injected into the liquid chromatography system (ExionLC AD, SCIEX, Framingham, MA, USA).

Chromatographic separation was performed using a Thermo Accucore™ C30 column (i.d. 2.1 × 100 mm, 2.6 μm). Solvent A consisted of acetonitrile/water (6:4, *v*/*v*) with 0.1% formic acid and 10 mM ammonium formate, and solvent B consisted of acetonitrile/isopropanol (1:9, *v*/*v*) with 0.1% formic acid and 10 mM ammonium formate. The gradient program for A/B volumetric ratio was set as follows: 0 min at 80:20, 2 min at 70:30, 4 min at 40:60, 9 min at 15:85, 14 min at 10:90, 15.5 min at 5:95, 17.3 min at 5:95, 17.5 min at 80:20, and 20 min at 80:20. The flow rate was 0.35 mL/min, and the column temperature was maintained at 45 °C.

Mass spectrometry was performed using ESI-QTRAP-MS in both positive and negative ion modes (QTRAP^®^, SCIEX, Framingham, MA, USA). The electrospray ionization source temperature was set at 500 °C, with a mass spectrometry voltage of 5500 V in positive ion mode and −4500 V in negative ion mode. MultiQuant 3.0.3 software (SCIEX, Framingham, MA, USA) was used to extract raw peak intensity data (*. wiff) from the Analyst 1.6.3 software (SCIEX, Framingham, MA, USA). The minimum chromatographic peak intensity for filtering was set at 1000 cps, with a minimum signal-to-noise ratio of 5, and a retention time deviation of less than 0.2 min. Based on the peak area of QC samples, substances with a detection rate of less than 50% and a CV greater than 0.3 were excluded. The qualitative and quantitative mass spectrometry analysis of the sample lipids was carried out based on MWDB, a database created by METWARE (https://www.metware.cn).

### 2.7. Statistical Analysis

Statistical analyses were performed using SPSS 26.0 (IBM, Chicago, IL, USA) and significant differences at *p* < 0.05 were analysed using Duncan’s multiple range test. Multivariate data analysis and random forest modeling were performed using Metaboanalyst 6.0 (https://www.metaboanalyst.ca).

Differential lipid screening employed a slightly modified version of the random forest model described by Liu et al. [18], setting the number of classification trees to 1000, randomly selecting one fifth of the data as out-of-bag data, and repeating the construction of each model 10 times in order to avoid the randomness of the results, assessing the performance of the model by the average OOB error rate, ranking the factors by the average variable importance, and progressively removing the factors with an importance of the last 20%.

## 3. Results and Discussion

### 3.1. Comparison of Tan Lamb Quality from Different Regions

**Figure 1:**
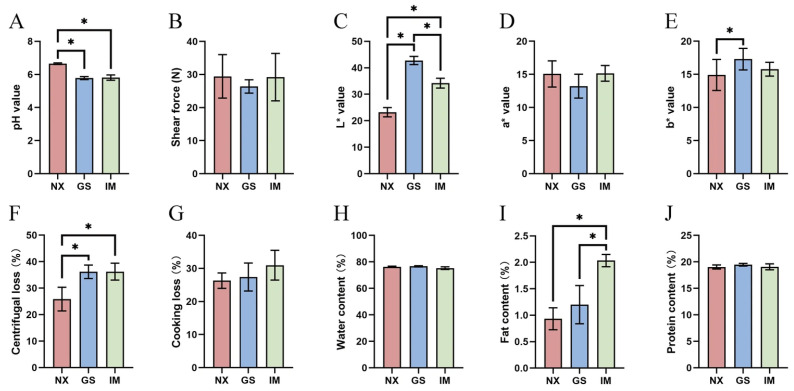
Comparison of the quality of Tan lamb meat in different regions. (**A**) pH value of Tan lamb from different regions. (**B**) Shear force of Tan lamb from different regions. (**C**) Lightness (L*) value of Tan lamb from different regions. (**D**) Redness (a*) value of Tan lamb from different regions. (**E**) Yellowness (b*) value of Tan lamb in different regions. (**F**) Centrifugal loss of Tan lamb in different regions. (**G**) Cooking loss of Tan lamb in different regions. (**H**) Moisture content of Tan lamb in different regions. (**I**) Fat content of Tan lamb in different regions. (**J**) Protein content of Tan lamb from different regions. * *p* < 0.05. (NX, Tan lamb from Yanchi, Ningxia; Gansu, Tan lamb from Jingyuan, Gansu; IM, Tan lamb from Ertokqianqi, Inner Mongolia; *n* = 6).

As shown in Figure 1A, the pH value of Ningxia Tan lamb is significantly higher than that of the other two regions (*p* < 0.05). There is no significant difference in the shear force of Tan lamb from different regions (Figure 1B). Regarding muscle colour (Figure 1C–E), Ningxia Tan lamb have lower L* and b*, there is no significant difference in a* values among the three regions (*p* > 0.05). By assessing the water-holding capacity of Tan lamb through centrifugal loss (Figure 1F) and cooking loss (Figure 1G), it is observed that the centrifugal loss of Ningxia Tan lamb is significantly lower than that of Gansu and Inner Mongolia (*p* < 0.05), while there is no significant difference in cooking loss among Tan lamb from the three regions (*p* > 0.05). Overall, Ningxia Tan lamb have better water-holding capacity. The unique forage resources and mineral elements in Yanchi may regulate the acid-base balance of mutton, potentially contributing to the higher pH value observed in Tan lamb. This elevated pH value, being distant from the isoelectric point of muscle proteins, allows the proteins to adopt a more extended spatial structure, enabling greater water retention. The moisture content on the surface of the lamb is positively correlated with its L*. The high pH value enhances the water-holding capacity of Yanchi Tan lamb, resulting in less moisture exuding from the surface. This could be one reason why Yanchi Tan lamb exhibits significantly lower L* compared to lamb from the other two regions [19]. The basic nutrition of Tan lamb from the three regions was evaluated based on water content (Figure 1H), fat (intramuscular fat, IMF) (Figure 1I), and protein (Figure 1J). Except for the IMF of Inner Mongolia Tan lamb, which is significantly higher than that of Ningxia and Gansu (*p* < 0.05), there were no significant differences observed in water content and protein content (*p* > 0.05).

### 3.2. Comparison of Tan Lamb Fatty Acid from Different Regions

The substances affecting the flavour of mutton mainly come from the fatty acids and their degradation products in the meat [20]. Table 1 lists the composition of fatty acids in Tan lamb from different regions. A total of 17 types of fatty acids were detected by GC-MS, including 8 saturated fatty acids (SFA), 4 monounsaturated fatty acids (MUFA), and 5 polyunsaturated fatty acids (PUFA). Except for lignoceric acid (C24:0), eicosenoic acid (C20:1n9), and eicosadienoic acid (C20:2), there were significant differences (*p* < 0.05) in the remaining 14 fatty acids among Tan lamb samples from different regions. Consistent with the research results of [13], palmitic acid (C16:0), stearic acid (C18:0), and oleic acid (C18:1n9c) are the main fatty acids in Tan lamb, closely related to the formation of the “mutton flavour” [21].

Comparing the total amounts of various fatty acids, Ningxia Tan lamb exhibit characteristics of low SFA, low MUFA, and high PUFA. The Yanchi area is rich in resources such as licorice and alfalfa [22]. These PUFA-rich pastures are consumed by sheep and deposited in the muscle tissue of Tan lambs. In addition, the high content of flavonoids and other antioxidants in Tan lamb meat protects PUFA, allowing it to accumulate steadily in the meat [23]. Compared to MUFA, PUFA, due to their more double bonds, are more prone to oxidation, promoting the generation of meat flavour [24]. Moreover, the proportion of unsaturated fatty acids (UFA) in Ningxia Tan lamb is also higher than in the other two regions. Specifically, the ratio of UFAs/FAs from high to low is as follows: Ningxia (0.531), Inner Mongolia (0.515), and Gansu (0.486).

Fatty acids, as one of the essential nutrients for the human body, also affect human health. Comparatively, polyunsaturated fatty acids are more beneficial to human health. The content of linoleic acid in Ningxia Tan lamb is significantly higher than in the other two regions (*p* < 0.05), playing a positive role in maintaining blood lipid balance [25]. In terms of arachidonic acid content, Ningxia and Inner Mongolia are significantly higher than Gansu, having a better effect on enhancing human immunity and combating cardiovascular diseases [26].

Dietary components and climatic environment might be important factors causing differences in the content and proportion of fatty acids in Tan lamb from the three regions. The combination of stall feeding and grazing provides greater opportunities for the intake of fatty acids in Tan sheep. Herbal plants are rich in polyunsaturated fatty acids, while grain feed might lead to higher saturated fatty acid content [1]. The natural grassland in Yanchi County, Ningxia, rich in Chinese herbs, provides more sources of polyunsaturated fatty acids for Tan lamb. The unique saline–alkali environment of Yanchi County may also be a key factor influencing the high proportion of unsaturated fatty acids in Ningxia Tan lamb.

### 3.3. GC-IMS Analysis of Tan Lamb from Different Regions

**Figure 2:**
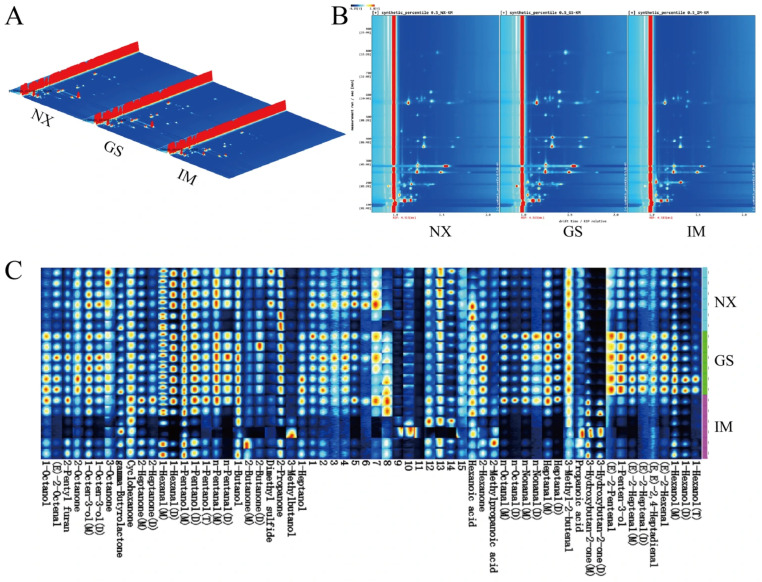
GC-IMS spectra of volatile components of Tan lamb meat in different regions. (**A**) 3D topographic plot. (**B**) 2D topographic plot. (**C**) Characteristic fingerprint spectra. (NX, Tan lamb from Yanchi, Ningxia; GS, Tan lamb from Jingyuan, Gansu; IM, Tan lamb from Ertokqianqi, Inner Mongolia; *n* = 6).

The sensitivity of GC-IMS to compounds such as aldehydes, ketones, and alcohols is useful in understanding the differences in volatile flavor substances among Tan lamb from different regions [27]. The results showed that 33 volatile compounds, including 11 aldehydes, 8 alcohols, 8 ketones, 3 acids, 1 ester, 1 furan and 1 sulfide, were detected in Tan lamb from the three regions. Figure 2A,B shows 3D and 2D topographic maps of volatile compounds in Tan lamb from the three regions. The results showed that the types of volatile compounds in Tan lamb from the three different regions were similar, but the signal intensities of the volatile components were different. In order to visualize the differences in volatile compounds in the samples of Tan lamb from the three regions, fingerprints were constructed using the Gallery plot plug-in in VOCal 0.4.10 software (Figure 2C). The existence of different forms of 1-Hexanol, (E)-2-Heptenal, n-Nonanal, and n-Octanal is due to the fact that when the concentration of the analyte is increased, in the ionization region of the ion mobility spectrum, two or three molecules will share a proton or an electron to form a dimer(D) or even a trimer(T), which migrates to the Faraday disk, respectively, and these different polymers are in fact a substance [28]. Compared with samples from other regions, the aldehydes in the Gansu Tan lamb samples had relatively high signaling responses, consistent with its primary precursor, α-linolenic acid, (E)-2-Octenal and (E)-2-Hexenal also exhibit high levels in Gansu Tan lamb, serving as key contributors to its grassy aroma. Additionally, n-Nonanal, Heptanal, (E)-2-Heptenal, and (E,E)-2,4-Heptadienal impart a fatty odor to the meat through the oxidation of lipids such as oleic acid and linoleic acid [29]. Feeding habits and feed composition influence fat oxidation and hydrolysis, determining the formation of aldehydes. Increased exercise enhances the body’s antioxidant capacity and promotes greater intake of antioxidant-active components, thereby reducing the oxidation of unsaturated fatty acids. This results in lower aldehyde levels in the meat of Yanchi and Inner Mongolia Tan sheep. In Inner Mongolia Tan lamb samples, 3-Hydroxybutan-2-one signal intensity was higher, and its main contribution was to provide butter flavor and creamy flavor, except for 1-Octen-3-ol and 2-Octanone signals in Inner Mongolia Tan lamb samples, which were relatively low accordingly. Among them, 1-Octen-3-ol is a degradation product of hydroperoxides of linoleate or linolenate having a mushroom aroma, which is useful for flavor formation of lamb. Overall, the results of GC-IMS fingerprinting showed that although the volatile compounds in the Tan lamb samples from the three regions were composed of similar kinds of volatile compounds, the contents of each substance were different and showed different flavor characteristics.

### 3.4. Reliability Validation of Lipidomic Data

The high stability of instruments is a crucial guarantee for the reliability of data. We assessed the reliability of the lipidomic data through the analysis of quality control (QC) samples. Five QC samples were prepared by mixing equal amounts of six Ningxia samples, six Gansu samples, and six Inner Mongolia samples. First, we analysed the total ion chromatograms of the QC samples in positive ion mode (Figure S1A) and negative ion mode (Figure S1B). The results showed that the retention time and peak intensity of the QC samples were consistent under both ion modes. The curves of the five QC samples highly overlapped, indicating that the mass spectrometry system had good signal stability. Subsequently, Pearson correlation analysis was performed on the QC samples. The |r| values among the five QC samples were all greater than 0.99 (Figure S1C), indicating high correlation among the samples and high stability during the detection process. Additionally, the five QC samples clustered at the center of the PCA plot (Figure S1D), further demonstrating the reproducibility and stability of the experiment. In summary, the reliability of the data in this study is confirmed by the consistency of the QC samples and the good intragroup reproducibility.

### 3.5. Comparison of Tan Lamb Lipids from Different Regions

**Figure 3:**
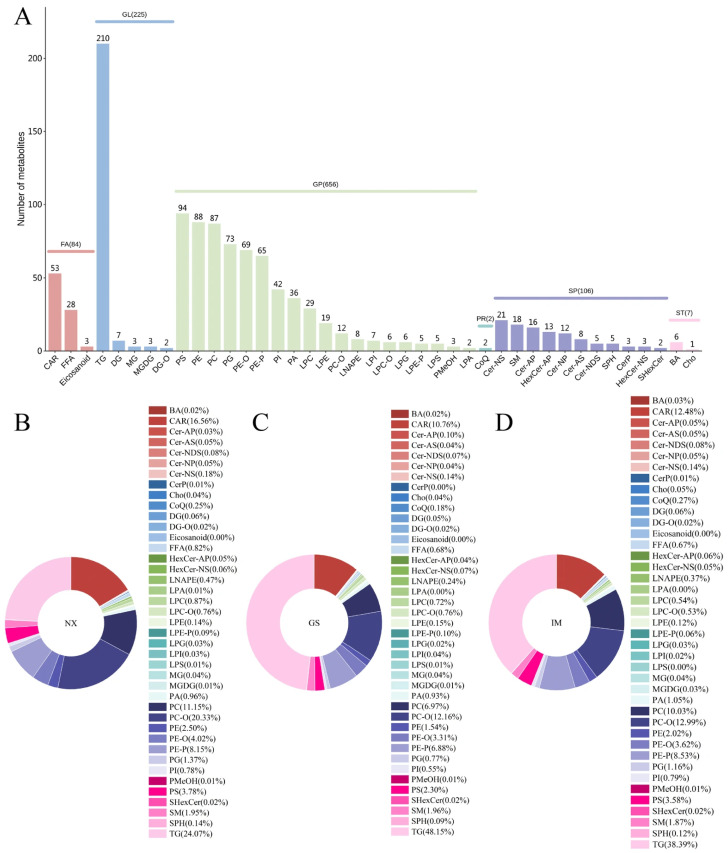
Lipid profiles of Tan lamb from different regions. (**A**) Lipid classes of Tan lamb from different regions. (**B**–**D**) Content of each lipid subclass in Tan lamb from different regions. (NX, Tan lamb from Yanchi, Ningxia; GS, Tan lamb from Jingyuan, Gansu; IM, Tan lamb from Ertokqianqi, Inner Mongolia).

As shown in Figure 3A, a total of 1080 lipids were identified from Tan lamb samples from the three regions, which can be categorized into six major classes: fatty acyl (FA) (84), glycerol (GL) (225), glycerophospholipid (GP) (656), isopentenolipid (PR) (2), sphingolipid (SP) (106), and sterolipid (ST) (7). Among these, GP is the most abundant lipid class in Tan lamb, followed by GL, with GP and GL together accounting for 81.57% of the total lipid classes. Further classification of all lipids resulted in 41 lipid subclasses, among which triacylglycerol (TG) (210), phosphatidylserine (PS) (94), phosphatidylethanolamine (PE) (88), and phosphatidylcholine (PC) (87) have a relatively large number of lipid types, affecting the abundance of lipid types in Tan lamb. TG is an important component of GL and plays an important role in energy storage, while PS, PE, and PC are important components of GP and maintain cell membrane structure and signal transmission. The rich variety of lipids in these four lipid subclasses also indirectly explains the high proportion of GP and GL in the total lipid composition.

Additionally, we analysed the content of various lipid subclasses in Tan lamb from different regions (Figure 3B–D), and the three regions showed some similarity in the proportion of major lipids. Specifically, CAR, PC, PC-O, PE-P, and TG are the five most abundant lipid subclasses in Tan lamb. This is similar to that found for the following major lipids in Hu sheep meat [30], where PC, an important phospholipid involved in cell structure, is widely present in animal tissues [31], and TG, as one of the main forms of fat, contributes to the high abundance of TG. Notably, there are differences in the proportion of lipid subclasses among different regions. For TG, the proportion in Gansu Tan lamb (48.15%) is higher than in Inner Mongolia Tan lamb (38.39%), which is higher than in Ningxia Tan lamb. Conversely, for PC-O, the proportion shows a different trend: Ningxia (20.33%) > Inner Mongolia (12.99%) > Gansu (12.16%).

We further compared the content of the top five lipid subclasses with the highest proportions in Tan lamb from different regions. As shown in Figure S2, the TG content in Gansu is significantly higher than in Ningxia (*p* < 0.05). For CAR content, Ningxia is significantly higher than the other two regions (*p* < 0.05). The differences in the content of PC-O, PC, and PE-P among the three regions are not significant (*p* > 0.05). Differences in dietary structure among regions affect the lipid composition and content in sheep meat. Besides the lipid differences directly caused by dietary intake [32], differences in active components [33,34] and mineral elements [35] in food may further influence lipid metabolism through regulation.

### 3.6. Screening of Differential Lipids in Tan Lamb from Different Regions

To further assess the differences in lipids among Tan lamb samples from different regions, we used a random forest model for screening. From the stepwise screening process, it can be seen that, similar to the screening of lipid markers in wines from different regions by Phan et al. [11], the classification error of the model initially decreases and then increases as the number of lipids decreases. The random forest model achieved a classification error of 0 with 94 lipids, indicating that these 94 lipids are important factors in distinguishing Tan lamb samples from the three regions. During the further lipid elimination process, as the bottom 20% of the least important factors were progressively eliminated from the 94 lipid features, the classification error of the random forest model remained zero throughout the reduction process until only 10 features remained. Further reduction in the number of lipids resulted in classification errors (Table S1). The 10 feature lipids identified (Table 2) are key to distinguishing Tan lamb samples from the three regions.

**Figure 4:**
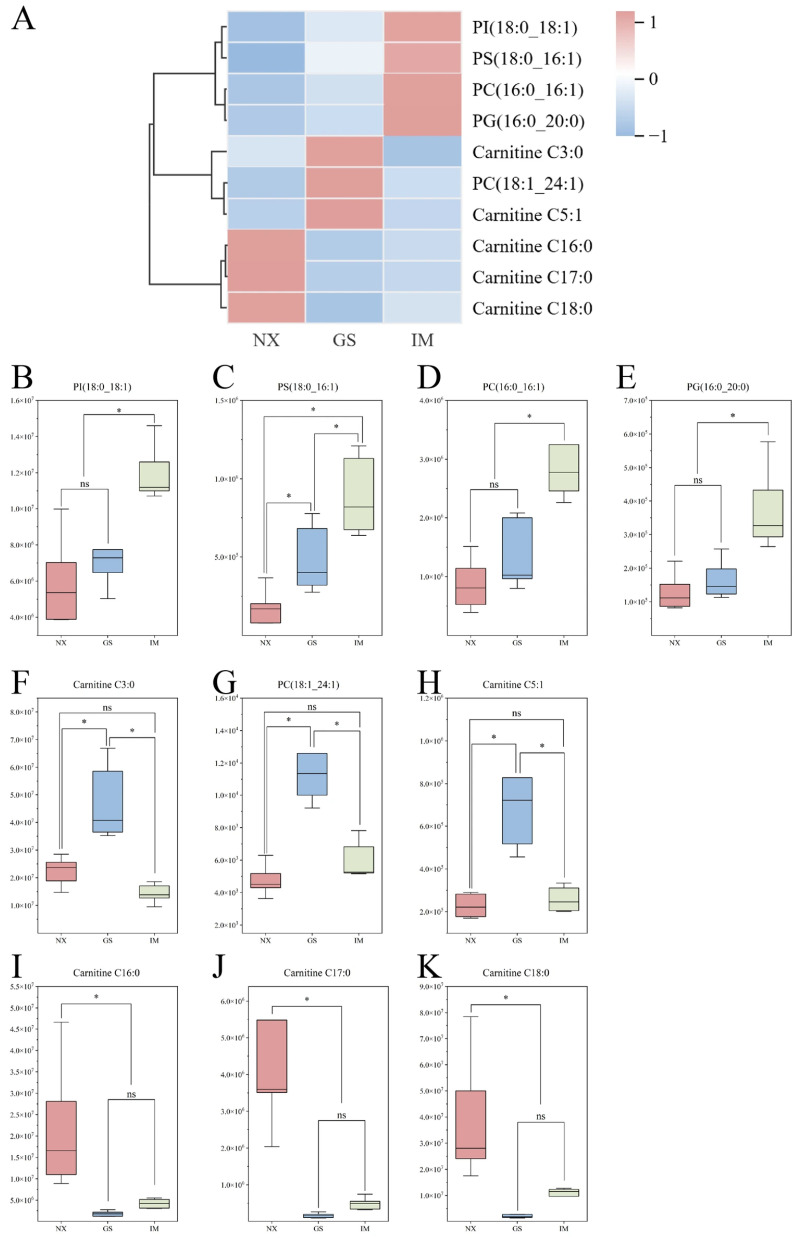
(**A**) Heat map of characteristic lipids of Tan lamb from different regions. (**B**–**K**) Box plots of differences in the content of each characteristic lipid in different regions. * *p* < 0.05, ^ns^ *p *> 0.05. (NX, Tan lamb from Yanchi, Ningxia; GS, Tan lamb from Jingyuan, Gansu; IM, Tan lamb from Ertokqianqi, Inner Mongolia; *n* = 6).

In addition, differences in characteristic lipids between Tan sheep from different regions were investigated using clustering heatmaps (Figure 4A). Heatmap colour represents the abundance of characteristic lipids, with red indicating higher and blue indicating lower abundance. The heatmap categorized the feature lipids into three groups. The first category includes PI (18:0–18:1), PS (18:0–16:1), PC (16:0–16:1), and PG (16:0–20:0), which show higher expression levels in Inner Mongolia Tan lamb. The second category consists of one PC and two types of Carnitines, showing higher expression levels in Gansu Tan lamb. The third category includes three types of Carnitines: C16:0, C17:0, and C18:0, which show higher expression levels in Ningxia Tan lamb. These findings suggest that the abundance of feature lipids may be a potential important factor influencing the differences in Tan lamb.

Moreover, we visualized the differences in the content of feature lipids in Tan lamb from different regions using box plots. The results show that the content of PI (18:0–18:1), PS (18:0–16:1), PC (16:0–16:1) and PG (16:0–20:0) is significantly higher in the Inner Mongolia region than in the other two regions (*p* < 0.05). The PS (18:0–16:1) content shows significant differences between the three regions, with the highest content in Inner Mongolia and the lowest in Ningxia. Compared to the Ningxia and Inner Mongolia regions, the content of Carnitine C3:0, Carnitine C5:1, and PC (18:1_24:1) is significantly higher in the Gansu region (*p* < 0.05). Furthermore, the concentration of carnitine C16:0, carnitine C17:0, and carnitine C18:0 in the Ningxia region is markedly higher than in the Gansu and Inner Mongolia regions (*p* < 0.05) (Figure 4B–K). These results are consistent with the observations from the heatmap (Figure 4A).

Differences in dietary composition leading to diverse lipid intake may be the primary cause of variations in lipid profiles. The pasture in the Yanchi region, rich in Chinese medicinal herbs, may enhance the intake of bioactive compounds such as acylcarnitines. Additionally, distinct soil and water conditions along with climatic factors may influence the growth rate and metabolic state of Tan sheep, potentially contributing to regional variations in characteristic lipid distribution.

### 3.7. Correlation Analysis Between Differential Lipids and Meat Quality of Tan Lamb from Different Regions

**Figure 5:**
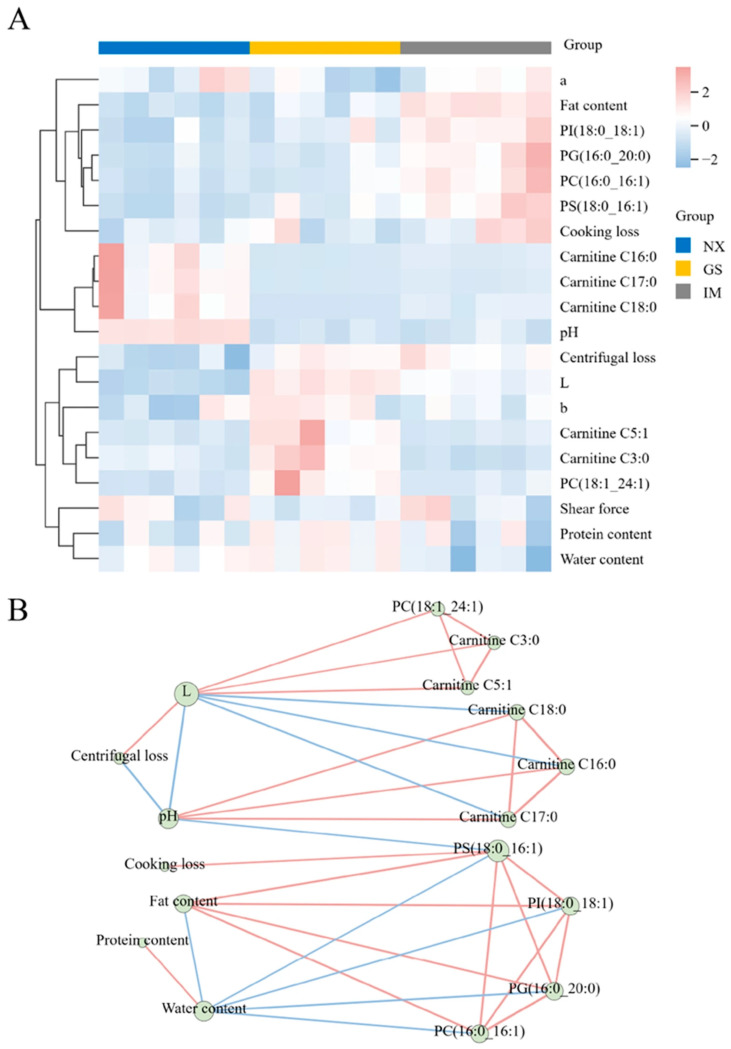
(**A**) Heat map of correlation between characteristic lipids and meat quality in different regions. (**B**) Network diagram of the correlation between characteristic lipids and meat quality in different regions. (Red line indicates positive correlation, blue line indicates negative correlation) (NX, Tan lamb from Yanchi, Ningxia; GS, Tan lamb from Jingyuan, Gansu; IM, Tan lamb from Ertokqianqi, Inner Mongolia).

Lipids have similar physiological and molecular characteristics, which create close relationships among them. As a major component of meat, they significantly influence meat quality [36]. The preliminary exploration of the relationship between lipids and meat quality of Tan lamb from different regions was conducted using heatmap analysis (Figure 5A). Next, we conducted a correlation network analysis to better visualize the correlations between characteristic lipids and meat quality. As shown in Figure 5B, most lipids exhibit high correlations, with more prominent interactions within the same lipid species. Glycerophospholipids (PS, PI, PG, PC) and fatty acyls (CAR) form dense clusters, similar to findings in yak milk by Li et al. [37]. The more prominent interactions within the same lipid species may be due to the similar physiological and molecular characteristics of lipid molecules of the same type, while the differences in identified characteristic lipids due to different detection subjects may be the primary factor contributing to the formation of distinct clusters.

Within the correlation network, meat quality indicators are aggregated into three parts. In the first part, centrifugal loss shows a positive correlation with lightness value, while pH shows a negative correlation with both. Centrifugal loss can indicate the water-holding capacity of meat; high centrifugal loss suggests poor water-holding capacity, leading to easier moisture loss from the meat [38], and possibly causing higher surface moisture content and thus higher lightness [39]. The pH value affects the water-holding capacity by influencing the solubility and water-binding capacity of proteins in the meat [40]. Under low pH conditions, increased protein solubility and structural changes lead to more moisture loss, which may explain the negative correlation between pH, centrifugal loss, and lightness.

Cooking loss exists independently in the second part. As another indicator of water-holding capacity during meat processing, it should show a positive correlation with centrifugal loss [41]. This conclusion is reflected by the bridging of PS (18:0–16:1) and pH value. The low correlation between centrifugal loss and cooking loss below the threshold set for plotting may be the main reason for their indirect correlation in the correlation network.

The third part includes three basic nutritional components: fat content, water content, and protein content. Water content is negatively correlated with the fat content and positively correlated with the protein content. With the total mass of the meat remaining constant, the substitution effect between substances leads to the phenomenon where one increases while another decreases. Water and fat are insoluble in each other, but protein can dissolve in water, which may cause the different correlations with water content.

Notably, in the correlation between meat quality and characteristic lipids, lightness shows different correlations with the content of various carnitines. Specifically, the lightness of meat shows a positive correlation with the content of Carnitine C3:0 and Carnitine C5:1, and a negative correlation with the content of three long carbon chains of carnitine (C16:0, C17:0 and C18:0). The length of the carbon chain may be the reason for this phenomenon; longer carbon chains in long-chain lipids make them more susceptible to oxidation, affecting the colour of the meat and making it look darker [42]. The homology of glycerophospholipids (PC (16:0–16:1), PS (18:0–16:1), PG (16:0–20:0), PI (18:0–18:1)) with crude fat may be the primary factor contributing to the positive correlation between the content of these four glycerophospholipids and the crude fat content. This also leads to a negative correlation between moisture content in the meat and the content of these four lipids.

### 3.8. Analysis of Characteristic Lipid Metabolic Pathways

**Figure 6:**
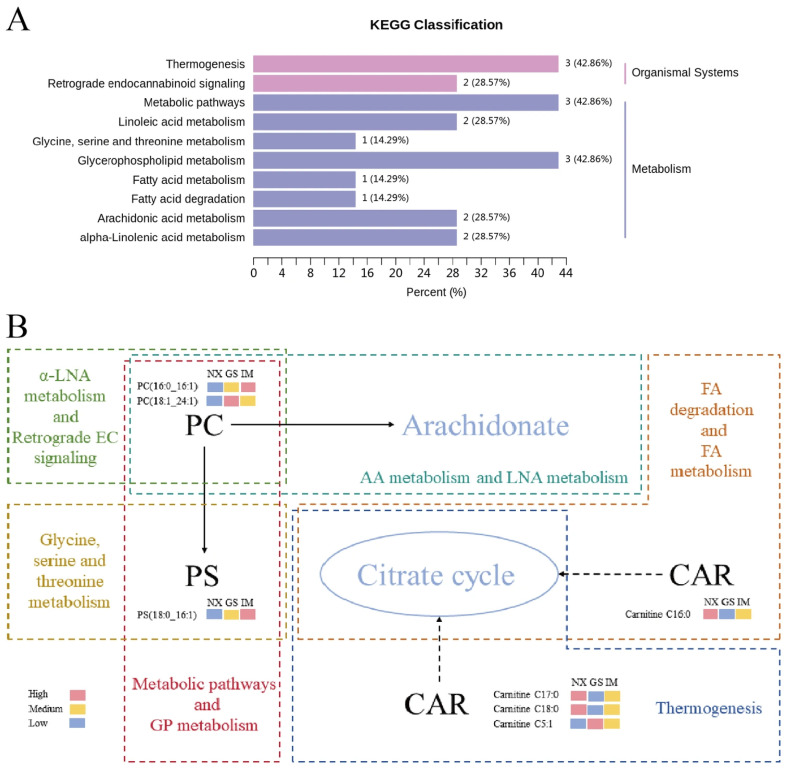
(**A**) Metabolic pathways of characteristic lipids of Tan lamb from different regions. (**B**) Relationship between the metabolic pathways involved in each metabolic pathway and the characteristic lipids. (NX, Tan lamb from Yanchi, Ningxia; GS, Tan lamb from Jingyuan, Gansu; IM, Tan lamb from Ertokqianqi, Inner Mongolia; PC, phosphatidylcholine; PS, phosphatidylserine; CAR, acylcarnitine).

Metabolic pathways are key to the differences in lipids within organisms. To elucidate the metabolic pathways related to characteristic lipids in Tan lamb from different regions, the 10 characteristic lipids identified were analysed for metabolic pathways using the Metware Cloud (https://cloud.metware.cn). As shown in Figure 6A, the characteristic lipids are primarily involved in 10 metabolic pathways related to organic systems and metabolism. Among all pathways, thermogenesis, metabolic pathways, and glycerophospholipid metabolism exhibited relatively high abundance. The differences in climatic conditions and dietary sources across different regions may significantly impact thermogenesis and metabolic biochemical reactions during the growth of Tan lamb, which could explain the observed phenomena.

Phospholipid metabolism is a crucial intracellular process, confirmed to be highly correlated with lipid changes during the growth of black morel [43]. In this study, it was found that 2 PCs and 1 PS were involved in metabolic pathways and glycerophospholipid metabolism. PC, as a substrate for PS synthesis, can undergo base exchange with serine under the catalysis of phosphatidylserine synthase [44]. As shown in Figure 6B, the 2 identified PCs also participate in four metabolic pathways: arachidonic acid metabolism, α-linolenic acid metabolism endocannabinoid retrograde signalling and linoleic acid metabolism. These PCs are crucial substrates for arachidonic acid synthesis in arachidonic acid and linoleic acid metabolism and can form arachidonic acid under the catalysis of phospholipase D [45].

Four CARs among the characteristic lipids were involved in three metabolic pathways. Carnitine C17:0, Carnitine C5:1 and Carnitine C18:0 all participated in the thermogenesis pathway, while Carnitine C16:0 was related to fatty acid metabolism and degradation. The formation and transport of acylcarnitine are critical for fatty acid oxidation, providing acetyl-CoA for the tricarboxylic acid cycle through β-oxidation, thereby playing a vital role in cellular energy metabolism [46].

## 4. Conclusions

This paper clarifies the differences in quality, fatty acids, and flavor among Tan lambs from different geographical regions and confirms the feasibility of using widely targeted lipidomics approach to explore the lipid characteristics of Tan lambs from these regions. Tan lamb from Yancheng showed higher pH, lower brightness and yellowness, better water retention, and more polyunsaturated fatty acids. Tan lamb from different regions showed different flavor characteristics, and lamb quality was closely related to lipid biomarkers selected by random forest. Thermogenesis, phospholipid metabolism, and metabolic pathways may be key factors affecting the differences in the quality of Tan lamb from different regions. This study complements existing knowledge on regional variations in Tan lamb quality and provides additional lipidomics data. It deepens our understanding of the quality characteristics of Tan lamb and the underlying lipid metabolic pathways, thereby aiding future efforts in identifying Tan lamb origins and controlling and enhancing meat quality. In the future, it will be important to provide more comprehensive evidence for the development of high-quality Tan lamb focusing on metabolites, proteins, and genetic factors.

## Figures and Tables

**Table 1:** Fatty acids contents of Tan lamb from different regions (g/100 g).

	NX	GS	IM
C10:0	0.004 ± 0.001 ^b^	0.008 ± 0.002 ^a^	0.004 ± 0.001 ^b^
C12:0	0.008 ± 0.003 ^b^	0.030 ± 0.002 ^a^	0.008 ± 0.003 ^b^
C14:0	0.081 ± 0.005 ^c^	0.251 ± 0.007 ^a^	0.089 ± 0.005 ^b^
C15:0	0.020 ± 0.004 ^b^	0.025 ± 0.004 ^a^	0.023 ± 0.003 ^ab^
C16:0	0.711 ± 0.009 ^c^	0.959 ± 0.010 ^a^	0.912 ± 0.009 ^b^
C17:0	0.085 ± 0.008 ^b^	0.053 ± 0.008 ^c^	0.101 ± 0.008 ^a^
C18:0	0.609 ± 0.011 ^b^	0.533 ± 0.010 ^c^	0.727 ± 0.010 ^a^
C24:0	0.005 ± 0.002	0.006 ± 0.002	0.005 ± 0.002
C14:1	0.003 ± 0.001 ^b^	0.009 ± 0.001 ^a^	0.003 ± 0.001 ^b^
C16:1	0.078 ± 0.004 ^c^	0.115 ± 0.002 ^a^	0.085 ± 0.006 ^b^
C18:1n9c	1.181 ± 0.011 ^c^	1.279 ± 0.003 ^b^	1.429 ± 0.013 ^a^
C20:1n9	0.003 ± 0.001	0.004 ± 0.002	0.004 ± 0.001
C18:2n6c	0.313 ± 0.002 ^a^	0.247 ± 0.002 ^c^	0.308 ± 0.002 ^b^
C18:3n3	0.004 ± 0.002 ^b^	0.015 ± 0.002 ^a^	0.006 ± 0.002 ^b^
C20:2	0.002 ± 0.001	0.002 ± 0.001	0.002 ± 0.001
C20:3n6	0.010 ± 0.001 ^a^	0.005 ± 0.001 ^b^	0.011 ± 0.001 ^a^
C20:4n6	0.132 ± 0.003 ^a^	0.089 ± 0.004 ^b^	0.135 ± 0.003 ^a^
SFA	1.523 ± 0.042 ^b^	1.864 ± 0.042 ^a^	1.869 ± 0.038 ^a^
MUFA	1.265 ± 0.014 ^c^	1.407 ± 0.004 ^b^	1.521 ± 0.006 ^a^
PUFA	0.460 ± 0.008 ^a^	0.358 ± 0.006 ^b^	0.462 ± 0.006 ^a^

a–c Different letters indicate statistically significant differences based on a Duncan test at a level of significance of *p* < 0.05.

**Table 2:** Characteristic lipids of Tan lamb from different regions.

9	Formula	Molecular Weight	Ionization Model	Lipid Category
Carnitine C16:0	C_23_H_45_NO_4_	399.33	[M+H]^+^	Fatty Acyls
Carnitine C17:0	C_24_H_47_NO_4_	413.35	[M+H]^+^	Fatty Acyls
Carnitine C18:0	C_25_H_49_NO_4_	427.37	[M+H]^+^	Fatty Acyls
Carnitine C3:0	C_10_H_19_NO_4_	217.13	[M+H]^+^	Fatty Acyls
Carnitine C5:1	C_12_H_21_NO_4_	243.15	[M+H]^+^	Fatty Acyls
PC (16:0_16:1)	C_40_H_78_NO_8_P	731.55	[M+COOH]^−^	Glycerophospholipids
PC (18:1_24:1)	C_50_H_96_NO_8_P	869.69	[M+COOH]^−^	Glycerophospholipids
PG (16:0_20:0)	C_42_H_83_O_10_P	778.57	[M−H]^−^	Glycerophospholipids
PI (18:0_18:1)	C_45_H_85_O_13_P	864.57	[M−H]^−^	Glycerophospholipids
PS (18:0_16:1)	C_40_H_76_NO_10_P	761.52	[M−H]^−^	Glycerophospholipids

## Data Availability

The original contributions presented in the study are included in the article and Supplementary Materials, further inquiries can be directed to the corresponding author.

## Supplementary Materials

The following supporting information can be downloaded at: https://www.mdpi.com/article/10.3390/foods14234046/s1, Figure S1. Reliability validation of lipidomic data. Total ion flow plots of QC samples in positive ion mode (A) and negative ion mode (B); (C) Pearson correlation analysis of QC samples; (D) Plot of principal component analysis of Tan lamb samples from three regions containing QC samples; Figure S2. Boxplots of the contents of the top 5 lipid subclasses with the highest proportions in Tan lamb from different regions. * *p* < 0.05. (NX, Tan lamb from Yanchi, Ningxia; GS, Tan lamb from Jingyuan, Gansu; IM, Tan lamb from Ertokqianqi, Inner Mongolia); Table S1. Percent out-of-bag(% OOB) ± standard deviation of lipid combination models; Table S2. Fatty acid standard curve.
