# Supplementary material for: Random Forest-Assisted Widely Targeted Lipidomic Reveals Differences in Tan Lamb Meat Quality in Different Regions
Source: Foods. 2025 Nov 26;14(23):4046. doi: 10.3390/foods14234046 (PMC12692676; doi:10.3390/foods14234046)
Supplement: Supplementary file 1 [file foods-14-04046-s001.zip › foods-3989638-supplementary.pdf]

Supplementary Information

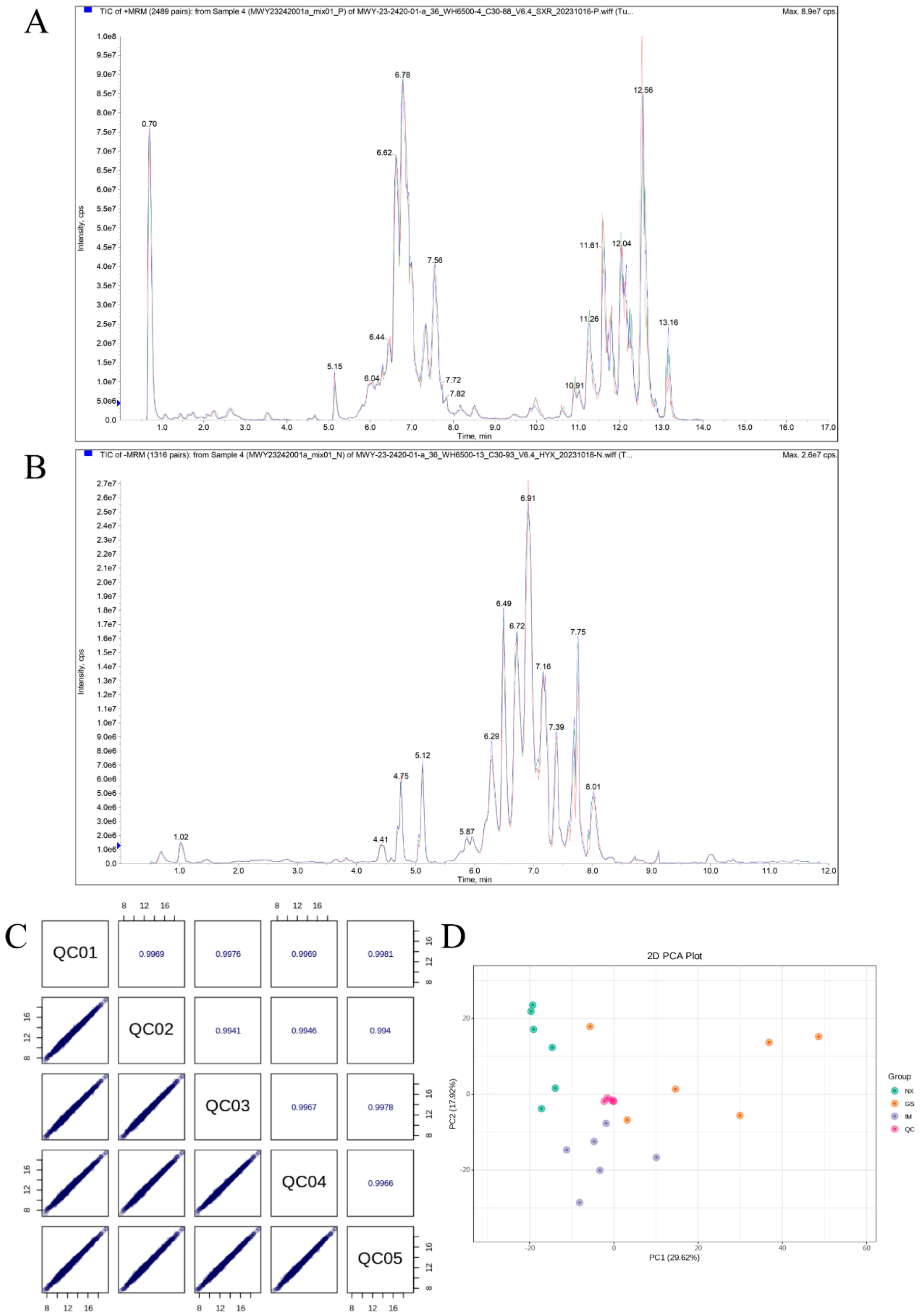

Figure S1. Reliability validation of lipidomic data. Total ion flow plots of QC samples in positive ion mode (A) and negative ion mode (B); (C) Pearson correlation analysis of QC samples; (D) Plot of principal component analysis of Tan lamb samples from three regions containing QC samples.

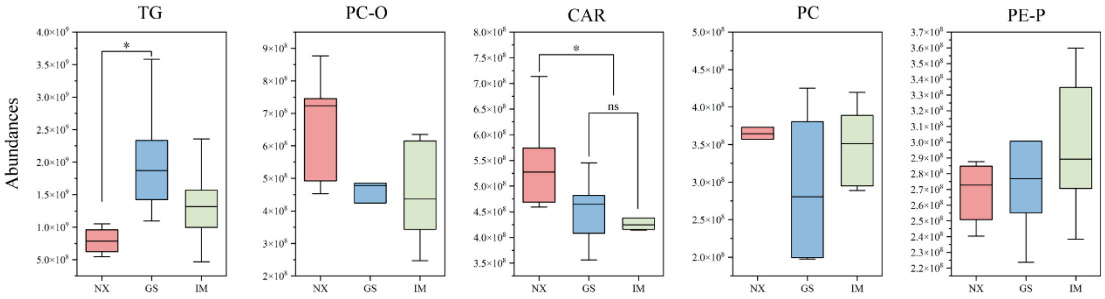

Figure S2. Boxplots of the contents of the top 5 lipid subclasses with the highest proportions in Tan lamb from different regions. $*p < 0.05$. (NX, Tan lamb from Yanchi, Ningxia; GS, Tan lamb from Jingyuan, Gansu; IM, Tan lamb from Ertokqianqi, Inner Mongolia).

Table S1. Percent out-of-bag(% OOB)± standard deviation of lipid combination models.

Number of Variables	% Out of Bag Error	Standard Deviation of % OOB
1080	23.88	7.46
864	20.00	2.69
691	17.22	5.80
552	9.44	3.56
441	6.11	3.88
358	1.11	2.22
286	2.78	2.78
229	5.00	1.67
183	5.00	1.67
146	2.22	2.72
117	1.11	2.21
94	0	0
75	0	0
60	0	0
48	0	0
38	0	0
30	0	0
24	0	0
19	0	0
15	0	0
12	0	0
10	0	0
8	6.11	3.88
6	2.78	2.78
5	2.78	2.78
4	3.34	2.72
3	4.45	2.22
2	7.22	2.54

Table S2. Fatty acid standard curve.

	Equation	R^2	LOQ (g/100 g)
C10:0	y = 86914.234747 x - 384.684220	0.996709614	0.002
C12:0	y = 205148.452432 x - 1555.503727	0.992492756	0.002
C14:0	y = 345535.544770 x - 3479.571962	0.997270414	0.001
C15:0	y = 464346.687562 x - 9784.860948	0.997535388	0.001
C16:0	y = 536126.179195 x - 10772.998079	0.993371995	0.002
C17:0	y = 64527.7860186 x - 2434.7685191	0.992576796	0.002
C18:0	y = 731927.296113 x - 26293.305107	0.996820364	0.002
C24:0	y = 654180.372343 x - 148638.546719	0.997313424	0.001
C14:1	y = 75283.842678 x - 762.672345	0.994658789	0.001
C16:1	y = 74321.543468 x - 2051.379955	0.993938806	0.001
C18:1n9c	y = 115068.383549 x - 4915.772381	0.990511134	0.002
C20:1n9	y = 80324.983249 x - 4132.884990	0.991735107	0.001
C18:2n6c	y = 234170.411382 x - 31318.898175	0.994798558	0.001
C18:3n3	y = 101877.669680 x - 5035.837143	0.992324163	0.001
C20:2	y = 212825.566321 x - 11626.131933	0.994239677	0.001
C20:3n6	y = 111906.605618 x - 6157.681662	0.993819622	0.001
C20:4n6	y = 11390.231979 x - 1029.353209	0.995208639	0.001
